# Event and Comment

**Published:** 1921-03

**Authors:** 


### DENTAL REGISTER

THE MAGAZINE OF DENTISTRY

Vol. LXXV | MARCH, 1921 | No. 3

### EVENT AND COMMENT

**A Classified Index for Dental Literature**

For several years there has been a longing on the part of dentists for an index, at least for the important articles contributed by the profession on the subject of Dentistry. We have no complete libraries in important dental centers where our literature could be consulted, even should one be disposed to look up what had already been written on any dental subject. It is therefore a matter of sincere congratulation that at last we now have the means by which any one can find out what has been written and could by making a proper effort get in touch with some of the few more or less complete books to be had on most dental topics. It is not possible to even understand what an avenue of approach to historic writing this will make possible. It will certainly make us acquainted with things done and give a new stimulus for a better literary grounding than is now possible. We are exceedingly grateful to all those who have labored so long to get this entering wedge, which we confidently believe will pry open new and surprising ideas, which ought to accelerate our development and help to stabilize the better forms of dental thought and action. The following abstract from an editorial in the *Journal of the National Dental Association* gives in the main the character of the work already done. and we hope every dentist will subscribe at once for a copy of the first volume:

"The American Institute of Dental Teachers has announced the publication of an index volume of five hundred and thirty pages, covering the periodical literature as published in the United States, England, Canada, Australia and New Zealand, for the five years, 1911 to 1915, and a second volume under way for the years 1916 to 1920. Each of these volumes will be in two parts; a subject index containing about twenty thousand items, titles arranged according to subjects; and an author index, containing about thirty thousand entries, including titles of books, papers, discussions, clinics, and biographies for each contributor. It is the intention to index the literature previous to 1911.

The index has been made on the plan of the Dewey Decimal Classification in use by most of our public libraries, by which all of the articles on each subject are grouped, regardless of the wording of their titles. There is a separate department for each branch of dentistry with as many subdivisions as necessary, with a list of articles grouped under each heading. It is, therefore, a very simple matter to find what has been written on any desired subject.

The index opens the door of the past and should strengthen the mind and hand of every practitioner for the future. Our literature reflects too little of the writings of those who have gone before. The constant restatements of facts indicate lack of acquaintance with previous contributions. The character of our journal articles should be an index of our professional achievement, yet there are few articles published today to which are attached adequate bibliographies.

One should not presume to write an article without acquainting himself with previous writings on his subject, yet it has heretofore been impossible to find what has been written. This has not been the fault of the individual contributor so much as the lack of development of the profession which would demand that our literature be made accessible.

This work has been done under the direction of Dr. Arthur D. Black, of Chicago. It has been financed by our dental colleges, societies, journals and a group of about fifty practitioners. The House of Delegates of the National Dental Association voted to assist with the preparation of the second index volume. It is not a publication for profit. Every dentist who is interested in the advancement of the profession should subscribe for a copy and should forthwith begin the accumulation of a dental library, if he has not already done so. The price of the index is $6. Orders should be sent at once to Dr. Abram Hoffman, 381 Linwood Ave., Buffalo, N. Y."

---

### UNVEILING OF MONUMENT AND MEMORIAL TABLET TO FREDERICK HENRY REHWINKEL, M.D., D.D.S., AT CHILLICOTHE, OHIO

---

On November 29, 1920, an occasion of unusual interest to the dental profession took place at Grandview Cemetery, Chillicothe, Ohio, in the unveiling of a memorial tablet to Dr. F. H. Rehwinkel, as a preliminary event to the opening of the fifty-fifth annual session of the Ohio State Dental Society at Columbus.

Pursuant to the arrangement, as announced in the program for the state society meeting, a number of dentists met at the Masonic Temple, and at 2:00 P. M., accompanied by about fifty members of the Chillicothe Commandery No. 8, Knights Templar, under a special dispensation to participate in the occasion, proceeded to the grave of Dr. Rehwinkel, where, after properly assembling, exercises took place.

Dr. Charles W. Mills, of Chillicothe, president of the Ohio State Dental Society, spoke as follows:

''Members of the Dental Profession and Fraters of Chillicothe Commandery: We have assembled on this occasion to do honor to the memory of one who in life enjoyed a national reputation in our profession for his literary contributions and zealous activities in advancing the standards of dentistry. His private life was an exemplification of the principles of Templar Knighthood, and for his zealousness in this order he received a deserved recognition of being chosen Right Eminent Grand Commander Knights Templar of Ohio, and in Scottish Rite Masonry was advanced to the thirty-third and last degree.

As dentistry and free masonry were the subjects in which he was best known to his generation, it was deemed most fitting for this occasion to have an address, on his activities as a Mason and one on his activities as a dentist.''

Assisted by Dr. L. E. Custer, of Dayton, Ohio, Master Charles William Mills, son of the chairman of the meeting, unveiled the monument and tablet, the inscription on which is here reproduced:

FREDERICK HENRY REHWINKEL, M.D., D.D.S.
June 15, 1825—June 8, 1889

President American Dental Association 1877; President Ohio State Dental Society 1869 and 1885; Member State Board of Dental Examiners 1868-1889; President Mississippi Valley Association of Dental Surgeons 1872; Captain Co. E., 37th Ohio Volunteer Infantry 1861-1862; 1st Commander A. L. Brown Post No. 162, G. A. R. 1881-1882; Right Eminent Grand Commander Knights Templar of Ohio 1887; 33° A. A. Scottish Rite, N. Masonic Juris U. S. A. His scientific and scholarly attainments; his moderation and self-control; his quiet dignity and indomitable firmness, made a lasting imprint in every field of his activity.

The Ohio State Dental Society; Rehwinkel Dental Society; Masons of Chillocothe, November 29, 1920

Dr. Mills then introduced Mr. John P. McCune, of Columbus, Ohio, thirty-third degree active and past grand commander Knights Templar of Ohio, who spoke of "Rehwinkel and Masonry," in which he paid a beautiful tribute to the character of Dr. Rehwinkel, his activities in masonry and his zeal for the institution.

ADDRESS OF MR. JOHN P. M'CUNE, COLUMBUS

Members of the Profession and Fraters of Chillicothe Commandery: In accepting the invitation to address you on "Rehwinkel and Dentistry," it was done with much timidity on my part, because I felt the inadequacy of words to express fully the reverence in which I have always held the name of Dr. Rehwinkel, and the inspiration his preceptorship was to me when I entered the dental profession.

As my address refers somewhat to his professional activities, I would ask those of the laity present to bear patiently with me through that feature of it.

Instances are indeed rare in the history of a human life where such honor and recognition are given to an individual almost a third of a century after he has crossed the Great Divide as we are now paying to the memory of Frederick Henry Rehwinkel.

It is evident that the flight of years has not dimmed the luster of his achievements in those fields that were benefited by his activities. Our gratitude to him finds us on this occasion responding to that noble impulse which since time immemorial has swayed the human mind to commemorate certain important events, or to do honor to the memory of some individual who has acquired certain fame in some line of activity.

Science and literature have added more imperishable names to the world's annals than any other endeavor, because these specialties contribute largely to human development, comfort, happiness, and those essentials upon which our progress and expansion depend.

The names that stand pre-eminent in dentistry were in the main not those whose whole energy concentrated in their chosen specialty, but those who have been men of recreative diversion, and by indulging the various bents of their minds have, through contact and association, rounded out to fuller possibilities and are accepted as better balanced and more widely informed men.

Few men have been so signally honored, both in the profession and out of it, none more modest in receiving recognition than Dr. Rehwinkel, and the tablet we have today dedicated to him shall mutely testify to coming generations that the name it bears was revered by his confreres, and the recognition inscribed thereon should be an inspiration to them for more than ordinary attainment.

Association with him as a student soon caused in me a profound reverence for him as a man of high ideals with a mind versed in infinite subjects, which afforded him valuable relaxation from the stress of a busy practice. For example, entomology was one of his many diversions, and in the early spring he eagerly welcomed the strange and varied forms of insect life, which he studied with the enthusiasm of a scientist. His interest in scientific subjects was not his only means of diversion from the necessary cares of life. He was a great lover of nature and appreciated her handiwork, which was so fully lavished on the environs of the city of his adoption—for Chillicothe is so situated in the valley, with the beautiful hills surrounding it and the streams enriching her fertile plains, that one who had chosen the spot in which to live felt fortunate indeed; while those of us who claim it as the home of our nativity are well-rooted to the place and feel a tender kinship for it—for the sounds and accents which haunt it; and the definiteness of early memories are inwrought with affection and kindly acquaintance, so that one ever returns to partake of the restful spirit which pervades the place. He loved the call of the wild, and field sports were one of his many hobbies. His bird-dog "Queen" received the same loving care as a member of his household, and her almost human intelligence seemed to bespeak a loyalty to her fond but dignified master in the pursuit of the one out-of-door sport which was perhaps to him the most alluring.

As an estimate of his attainments by one of his confreres in the dental profession—in the discussion of a paper read before the New York Odontological Society, April 17, 1877, on "Devitalized Teeth and Their Management"—Dr. W. H. Atkinson, of New York, in his time dean of the dental profession, paid him the following tribute in opening the discussion:

"This is one of the very few papers that come before us entitled to or able to bear severe criticism. The gentleman being well learned, is much more likely to lead us astray when he is in error, because of his clearness of statement where his statements are true, for the reason that our confidence is gained by the clear coincidence of our own judgment with the main statements in the paper; therefore we had better be a little cautious about accepting the whole.—[*Dental Cosmos,* XIX (1877), 455.]"

In this paper he advocated the same conditions we are striving for today—those that will make necrotic tissue compatable with the living.

Touching on his technic in root-canal work, I have recently received a letter from Dr. Howard Raper, a native of Chillicothe, now of Indianapolis, in which he mentions several teeth, the roots of which had been successfully filled by Dr. Rehwinkel, as shown by an X-ray examination.

At a meeting of the American Dental Association in Chicago, 1877, as a report of the Committee on Pathology and Surgery, he read a paper entitled "Causes and Conditions Attending the Formidable Disease of the Gums and Root Membranes and Alveolar Process Known as Pyorrhea Alveolaris." In that paper he endeavored to establish "that pyorrhea may and does exist independently of foreign deposits, and that it does occur in the cleanest of mouths, and must, for its origin, depend upon other than mere local causes'' (Transactions of the American Dental Association, 1877; *Dental Cosmos,* 1877, p. 572).

In Black's *Special Dental Pathology* (1915) reference is given to the above paper as follows:

''Dr. Rehwinkel's paper, in 1877, was by far the best writing upon this subject up to that time. It abounds in inquiry rather than in the discovery or announcement of the principles of pathology. It is rich in reference of both American and European authors. Although it set a landmark in the name to which it gave origin, it did little to advance our knowledge of the disease. No hint is given of the pus pocket as such, or as a distinct entity in the pathology of the peridental membrane; the nearest approach to this idea is the mention of the peculiar form of calculus which occurs on the sides of roots where the membrane has been destroyed, and states it is sometimes different from salivary calculus; states many suppositions regarding systemic conditions as causative factors in inducing disease of this tissue.''

In the *Dental Cosmos* for 1875 (p. 278), under ''Hints and Queries,'' a condition of the gums is described, and a reply signed ''J. T.'' states that the disease as described indicated it to be ''pyorrhea alveolaris'' of the French writers. Without inquiry as to the origin of the term now so familiar to the laity, full credit must be given to Dr. Rehwinkel for introducing the term into American dental literature and having called the attention of the dental profession to its seriousness.

Time did not permit the compilation of a complete list of his contributions to literature, but his writings were lucid and instructive and of great benefit to the profession.

A skillful operator, careful student, a strong advocate for efficiency in all its branches of practice, Dr. Rehwinkel was classed with and enjoyed the sincere friendship of such stars in the dental firmament as Atkinson, Taft, Watt, Butler, Keely, Webb, McKellops, Palmer, Black, and scores of other contemporaries.

A man of high ideals, he was ever alert to raise the standard of his chosen profession. He was active in securing the passage of the first dental law in Ohio in 1868, defended it when assailed, and served continuously on the State Board of Examiners, which this law called into existence, from 1868 until his death, when he was succeeded by Dr. L. E. Custer, of Dayton, Ohio.

Graduating from the Baltimore College of Dental Surgery in March, 1855, he prepared a graduating thesis on "Causes of Dental Caries." In this class of twenty-seven graduates are found the names of F. J. S. Gorgas, H. McG. Grant, and others whose names became well known throughout the dental world.

His activities in the Ohio State Dental Society were as varied as the officers and committees would permit: Charter member, 1866; second vice-president, 1868; president, 1869 and 1885 (being the only person twice honored with this office); member Board of Examiners, 1868 to 1889, inclusive; Board of Directors, 1888 and 1889; Committee on Membership, 1866, 1867, 1868, 1887, 1888; Committee on Ethics, 1868, 1869, 1872; Executive Committee, 1874, 1875, 1876, 1878, 1879, 1882, 1884; Committee on Dental Law, 1881, 1885, 1887, 1888; Committee on Patents, 1871; Committee on Constitution, 1885; Clinic, 1886 on "Herbst Method of Filling." He served as president of the old Mississippi Valley Association of Dental Surgeons in 1872. In national dental affairs he was no less active. Elected president of the National Dental Association in 1877; secretary of the section of dental and oral surgery at the International Medical Congress in Washington, D. C., in 1887, and president of dental and oral section,

American Medical Association, and would have presided at Newport, R. I., June 25-27, 1889, had he lived.

In recognition of his dental activities, one of our most active of the component societies was christened in his honor as the Rehwinkel Society, at its organization September 18, 1910. He recognized early in his dental career the important and sustaining influence of association with members of his profession, for in them he found a community of interests which he could not resist. His membership in the various dental associations brought him in contact with men who were an inspiration to him, and he gave his hearty support to everything pertaining to the welfare of his profession. Of his contemporaries, or predecessors in Chillicothe, none, to my knowledge, were members of dental societies, or, if so, failed to get the stimulation that would give them anything more than a local prominence. The record of the results of Dr. Rehwinkel's activities are a strong contrast to those of the dentist, who, ostracizing himself from his professional associates, sinks into the proverbial rut which leads to the ante-mortem oblivion.

Next to his profession, free masonry was closest to his heart, and in this he advanced to the highest grade—the only member of our profession having been honored by the office of Right Eminent Grand Commander Knights Templar of Ohio, and the first of three who had attained the thirty-third degree Ancient and Accepted Scottish Rite. The other two, also deceased, were Drs. Jas. A. Stipp, Toledo, and Charles R. Butler, Cleveland.

In the civic affairs of Chillicothe he was well informed, but owing to his inclinations his activities were naturally along educational lines. He was for several years a member of the Board of Education, serving as president of that body in the early eighties. Throughout his professional career he maintained that a high-school diploma should be the credential for admission of students to dental colleges, and it is of interest to note that the dental law in Ohio, making this obligatory, became operative thirty years after his death. It may truly be said of him that some of his professional ideals were in advance of his time.

In enumerating the activities of Dr. Rehwinkel, it may not be amiss to include some biographical notes that have been obtained from various sources:

From the church register of Neuenhausen, Celle, Hanover, Germany, is found that Frederick Henrich Rehwinkel was born June 15, 1825, and was christened July 17, 1825, in the house of his father, Yohann Heinrich Rehwinkel, living in the part of Celle now known as Altenhausen. The family of Rehwinkel still living in Celle have no record or traditional memory of the man whom we honor today.

When about twenty-three years of age, he was graduated from a homeopathic medical college in his native country, and because of the political troubles in Germany he emigrated to America in 1848. One of the number of young men who came to this country at this time, and who was frequently mentioned by Dr. Rehwinkel, was Carl Schurz, who became a prominent member of our National Congress. A younger brother, to whom the doctor was greatly attached, having been stricken with tuberculosis, and to whom a sea voyage had been recommended, came with him. They arrived at Baltimore, but were advised to go farther south and went to Mississippi, where the invalid brother died a few months later.

Dr. Rehwinkel began the practice of medicine in Natchez, Miss., and afterward came to Cincinnati, Ohio, where he associated himself with one Dr. Ehrman, and they did much work during the epidemic of cholera there in 1849. Being advised of a promising opening in Chillicothe, Ohio, he located there in 1850, but not meeting the success he anticipated he moved to Portsmouth, Ohio, in

1851, where he associated with Dr. Zimri Hussey. In November, 1851, he married Mary K. Jones, of Chillicothe (who was born in 1839 and died in 1912). To them one daughter, Cora, was born, who still survives.

In December, 1852, he returned to Chillicothe, and in 1853, finding his tastes and inclinations more in harmony with the practice of dentistry, he began the study with Dr. Joseph Sanford, a pioneer dentist there, who died in 1864.

In 1854 he entered the Baltimore College of Dental Surgery. He often referred to the cordial greeting given him by Chapin A. Harris, dean and founder of the school, when the latter learned that he came from Chillicothe, only twenty miles from Bainbridge, where Harris' brother John had practiced medicine and where he and James Taylor, founder of the Ohio College of Dental Surgery, had both received their first instruction in dentistry. After graduation he returned to Chillicothe, where he soon built up a choice clientele, and practiced continuously until his death, June 8, 1889.

Though of foreign birth, his patriotism for the country of his adoption must not be omitted. The Thirty-seventh Ohio Volunteer Infantry was the third German regiment raised in Ohio and was recruited principally among the patriotic Germans of Cleveland, Toledo and Chillicothe. Its organization commenced under the second call of Lincoln for 300,000 men, in August, 1861; by October 1 seven full companies had reported, and Col. E. Siber, an accomplished German officer, who had seen active service in Prussia, was selected commanding officer. Dr. Rehwinkel having assisted in organizing Company E of the Thirty-seventh, was elected captain. After a short term of service, principally in the Kanawha Valley, West Virginia, he was ordered home on sick leave, and on returning to his regiment he was so injured by the overturning of a stagecoach near Gallipolis that he was obliged to use crutches for a year and had to retire from military service October 1, 1862.

During the excitement in southern Ohio, due to the raid of the guerilla chief, John Morgan, in 1863, he was colonel of a regiment of raw militia encamped on the land now bounded by Paint, Fifth, Caldwell and Seventh streets in Chillicothe. Some of his own troops burned the bridge over Paint Creek, then but about seven inches of water, and started the cry, "Morgan is coming!" He failed to come, however, and a few days later Dr. Rehwinkel marched his regiment from Athens, Ohio, to Chauncey, on the road to Ironton, and back, a distance of more than thirty-five miles, in one day. The weather was hot and the troops that succeeded in reaching Athens that night realized that they had covered some territory.

His relation to the Grand Army organization was to him a source of pleasure and pride, and from them the following record has been kindly furnished by A. L. Brown Post No. 162: Enlisted September 1, 1861; registered October 1, 1862; captain Company E, 37th O. V. I., 1861-62; mustered into G. A. R., December 2, 1881; first commander of the post, 1881-82. In small towns there prevails a spirit of comradeship which is not possible in the city. Perhaps by closer association men learn to know one another more intimately, and here and there we find groups of friends of more or less prominence in the life of the place.

Here in Chillicothe at the time Dr. Rehwinkel was advancing in years, but still active in his profession, was a coterie of men who were held in high regard by the younger generation. They represented the various professions, the mercantile pursuits, and others of some literary attainment and minds broadened by extensive travel. In fact, few other towns could boast of the type of men of such distinguished demeanor as were residing in Chillicothe at the time referred to. It was the custom of these gentlemen to stop occasionally at one of the stores opposite the old post-office on Second street for discussion of current topics. There was no prearrangement in this, simply "drop in," purely incidental meetings, but perhaps the more enjoyable by reason of this. It was not town gossip that brought them together, nor a matter of simply idling away the time; it meant a very real companionship, an honest exchange of ideas and views on the issues of the day. Among these men were counted many who meant much to the real life of the town and who were not only prominent in the home circles, but who had made names for themselves in the wider fields of activity, and among them was the man whom we honor today, whose interest in all matters of this kind was keen and intelligent.

Dr. Rehwinkel was a big man among men, a personality which dominated, an intellect which attracted and commanded. He had a keen sense of right and wrong, appreciating always the motive prompting an action. He was generous, friendly, sympathetic, kind-hearted, and ever ready to help any who were in trouble. He had a sincere faith in things spiritual, believing that the life beyond held the fulfillment of all earthly promise, the completion of work begun.

My personal estimate of the man whom we honor today is that his eminence as a dentist, his usefulness as a citizen, his character as a man secured for him an enviable reputation; while the frankness, generosity and nobleness of his heart won the lasting love of all who knew him.

Like flower-bloom and fragrant breath,
His life survives the touch of death.
So we turn from his grave with eyes undim,
To greet the new-born day—and him.

ADDRESS OF A. F. EMMINGER

Sir Knights, Friends, Brothers: It is not with a feeling of pleasure that I respond to this call to make a few remarks upon this memorable occasion, in honoring the memory of our departed brother and friend, but with a deep sense of sorrow. I deem it a great privilege.

It was my good fortune to know the deceased intimately, perhaps more intimately than any man in the profession of dentistry. From the first day of our meeting there seemed to spring up a mutual feeling of friendship and love. I was attracted to him and he seemingly to me. Our likes and dislikes seemed to run in parallel channels.

He loved nature and lived close to it. He was passionately fond of field sports, the rod, dog and gun.

It was our custom each year to spend the hunting season in the field with dog and gun. He maintained a kennel in Chillicothe (bird-dogs and beagle hounds) and I one in Columbus. On Friday night it was my custom to run down to Chillicothe. Saturday was spent roaming over the hills and through the valleys about his home town. Sunday was spent in social intercourse. If Sunday was bright and the sun shining we would take a walk and frequently find ourselves on Grandview Cemetery Hill, on this bluff where his body now lies cold in death, overlooking Paint Creek Valley. He would exclaim: "Em (as he called me), have you ever seen anything more beautiful?" The following Friday night he would run up to Columbus, and on Saturday we would repeat our program. This continued throughout the hunting season.

Brother Rehwinkel was a big man among his fellowmen, a man of high ideals, a man of scholarly attainments, a man who had a high conception of right and wrong and who wanted to be considered a right-minded man, always looking upon the bright side of life. He had a keen sense of humor, could tell a good story and enjoy a good story, knew where the laugh came in, and was always doing his part in the domain of amusement and entertainment. He was a formidable adversary in debate

—quick at repartee, caustic when occasion required. He despised hypocrisy and deceit; if he was your friend you knew it, and if he were against you, you knew it—no middle ground.

In public life he would have been a leader and would have gone to the top, as was evidenced in his Masonic career.

All in all, Brother Rehwinkel was a big, brainy man, of fine, high attainments and tender-hearted as a child. He was attracted and attached to the younger men in the profession, always ready and willing to extend the helping hand and encouragement.

Well do I recall when in New York City, attending the national meeting in 1881, Dr. Rehwinkel, Dr. George W. Keely and myself were walking down one of the principal thoroughfares and I had remarked "how they happened to cast their *lots* in small places or towns," when a funeral procession passed by. Dr. Rehwinkel took me by the arm and said: "Look at that, do you know who it is? That is why I am too old to change, and I have the consolation of thinking when my time comes everybody in Chillicothe will know who it is, and as the funeral procession passed down the street they will stand on the sidewalk and say, 'There goes poor old Rehwinkel.'" I looked at him and there were tears in his eyes and in mine, too.

In my early professional career he was my ideal and model and I felt I was a better man for my acquaintance and associations with him. Peace be to his ashes.

Mr. Richard Enderlin, of Chillicothe, representing the Grand Army, spoke of Dr. Rehwinkel's activities in the Civil War, eulogized his patriotism, and made a masterful plea for loyalty to our flag and our country.—*Journal N. D. A.*, February, 1921.

### THE VALUE OF CO-OPERATION BETWEEN THE DENTIST AND HIS BANKER

If there is any one thing that I do not like to do, it is to attend a lecture by some one who has no message. Words without purpose seem able to string themselves together in endless processions. But I do believe that I have a message for you tonight.

I would like to plant a "seed" thought, a basic idea—one that I know is capable of growing in the soil of your every-day lives. I should like to give you some thoughts that will point the way to greater business and personal happiness and contentment. Right-thinking men can not be satisfied with their work or their lives if they are neglecting any opportunities for improvement.

This matter of the relationship between the professional man—the dentist—and his banker is full of neglected opportunities; often, where the banker and the dentist are close friends, socially.

How can the dentist co-operate with the bank? Obviously, he can keep his cash balances with his bank, and there are other ways, but I do not think the dentist's co-operation with his banker is as lax, or as far from satisfactory as is the banker's offer of co-operation to the dentist.

The subject of the "banker's co-operation" is the one which I wish to discuss. I present this side of the case because a correct attitude on your part will help your banker to see his duty more clearly, and because I want you to know just what services the banker should afford you.

"Co-operation" means to operate together, or to operate with each other—it means mutual service.

My experience with institutional service has not been entirely satisfactory, particularly where it has been advertised as available, say, for example, where I bought my automobile. It apparently meant that goods would be sold to me by affable and courteous salesmen, that exactly the articles which I purchased would be delivered promptly, and that afterwards, no matter how angry I might get because the performance of the machine which I purchased, the service department would grin and forbear. Such is advertised service! A service organization is all right in its way—the same way that honesty is right—but service in an automobile has got to be built into the car!

Gentlemen, you are not selling gold crowns, nor are you working at so much per hour, but you are selling years of comfort and freedom from disease!

Away up in the Alps in Switzerland lives an old Swiss watchmaker; his workshop is in his kitchen; he is a gruff, unyielding old gentleman; he lacks tact, courtesy; he will not promise a watch by Saturday night; he is not prompt, but he builds the most exact and serviceable watches made in all Switzerland. True, he would be happier if he had a sunnier disposition—but the point is, he serves the world well. How? By the exercise of his wonderful technique! *Technique,* that is the word. Your value to the world is in your fingers, plus your brain—technique.

Gentlemen, so is the banker's value to you to be measured by the exercise of his technical skill in problems common to both of you, but in which his experience gives him better judgment and skill.

Now, let us see how bankers, generally, are exercising their technique, or I might say, using their talents, for the benefit of their community.

Let me warn you that probably seven out of ten bankers will either disagree with me as to their responsibilities or will refuse to comment on my remarks because the entire discussion is over their heads.

Banking today is not a business; it is a profession, the same as dentistry, law, medicine or engineering. A banker today has no business in business. He should be governed by as strict a code of ethics as in any other professional group. The banker who says that credit is a merchandise like clothing, to be sold to the highest bidder, is wrong. The banker's money is not his own to give to whom he chooses; it belongs to the community and should be used only for the benefit of the community.

Let me give you a parable! A certain bank had as clients five clothing stores; all were in good credit and prosperous. A sixth clothier came to town. He was of the type known as a "mixer"; a good sales merchant, but a poor business man. His new store prospered at first, so he soon borrowed money from his bank to add to his stock and to improve his store front. He used credit whenever it was allowed him, and finally, as is usual in such cases, his creditors shut down on him, and the bank put a man in charge. The new manager cut prices in half, added a stock of shoddy clothing, put up big red sales signs and proceeded to abstract the bank's loss from the pockets of the community. Not only that, but this sale so disorganized the clothing trade that two other of the bank's depositors who owned clothing stores failed. *They* never suspected that away down deep their failure was the banker's fault.

But, you say, how could the situation be avoided?

By the use of better banking technique. In this case an investigation of the antecedents of the new clothier would have disclosed his weakness. Our technique says that you do not know a man or his organization until you know his antecedents, reputation, character, efficiency and service rendered to the community.

I said that a banker, ethically, should not be actively in business. I ask you, if I operated a wholesale hardware enterprise, how could I give the best of my ability to my competitors who would be depositors in my bank; also, how could I help but use in my business information gleaned from them; furthermore, if I were both banker and merchant and my business as a merchant required my attention at critical moments, would the clients of my bank in a time of crisis be likely to find my skill available?

If I had a case at court, I should expect my attorney to put his "feet in my shoes"—give me all his time and skill—would I not expect him to give me half of his attention for one fee and all of it for another. Gentlemen, ethically, a banker is in the same position as the lawyer!

Another matter. If you went to your lawyer for advice and he suggested than a lengthy suit be entered because of the larger fee he could obtain from you, would you be satisfied? While the amount involved is by no means so great, is not the principle the same when a banker buys certain investment securities that he may sell them to you at a profit when there are other securities on the market which would much better fit your particular financial situation?

When a bond broker approaches you with information, you buy with your eyes open.

A banker, in my estimation, should not advise upon investments when he has profits at stake. The investment departments in my institution does not buy any securities for resale, but satisfies itself with analytical questions to assist in purchases made for the bank's permanent investment and for its clients.

A good service for a bank to afford is one similar to that offered by the advertising agencies.

When some magazine tries to sell an advertisement to the bank, I refer them to our advertising agents, who thoroughly check up the magazine, its circulation, its policy, etc. They make a report back to me upon the advisability of placing advertising in that particular medium.

A dentist should use some bank for a similar purpose in checking investments, and all banks, in my estimation, should be ready and capable of accepting such commissions.

Every bank should have a department of analysis and statistics capable of an independent and unbiased survey of any situation, whether it be to the credit of an individual, the purpose of a promotion enterprise, the progress of a manufacturing concern, or the advisability of an investment. Many banks have such a plant for the analysis of their internal problems. In every case these facilities should be freely at the disposal of the bank's depositors, whether great or small.

The experience of the banker, his insight into every kind of business, should be used like a doctor's experience on the inside of men—for the benefit of humankind generally.

My experience is that he profits most who serves best. *The more I give from my experience, the more I have and the more I have to give.*

I remember illustrating this truth to one of the young ladies at the bank. As I approached the bank one day, I found myself walking beside this certain young lady. She was smiling and apparently was very happy. I said, "You are very happy today." "Oh, yes," she replied, "I am. I have just purchased the most beautiful hat I ever saw!" "Oh," I replied, "I am also an admirer of beautiful millinery, let me see your hat, and then I can rejoice with you." *But* she would *not* open the bag!

I took a $20 bill from my pocket and said, "If you should find a bill like this would you be very happy?" "Yes, I wish I could find one," she said. "But," I replied, "for you to find one some one would have to be made sad at its loss." So it is with material things, but in mental and moral matters it is different. With them *to have you must give away* and the more you give the more you have. "Now," I said, "you try the experiment. If your hat pleases me, I shall be happy, and your happiness will be increased."

So she opened the bag.

It was a beautiful creation for a hat. I was glad to see her use such good taste in the selection of color, size, shape, and at last, but not least, modesty, in both style and price.

The exhibition of the hat touched the real Aladdin's lamp; there sprang into existence a greater stock of happiness than there had been before.

Gentlemen, I find this pleasure, this creative ability in my day's work. Let me ask you, Do you?

Once I visited a great gold mine; I was very much impressed with the great stamp mills; they ground great boulders into powder! The 2,000-foot shaft was a marvel of engineering. The elevators, the ore cars, the miners with their drills all cried aloud of a great genius for organization, so clocklike did everything work.

Finally, I had seen everything. "Where is the gold; I want to see the pure gold," I said. "Oh, you can not see that here," replied the superintendent; "come over to the little smelter." So in the quiet corner of a little assaying office far from the busy mine I saw *pure gold.* Without that little trickle of pure gold, the great stamps would stop, the men would go away, and the shaft would soon fill with water.

It is the same everywhere. All of our commotion, our wonderfully equipped offices, great buildings, immense wealth, dignified personalities—all are useless and of no value except for the services we render in some quiet corner conference.

I value my power to achieve. Power to accomplish is what we all want.

I shall never forget the story of the old miner—a desert rat—who, after a lifetime of wanderings and toil, found *the mother lode—the very place where gold grows.* It was in the pocket on the side of a desert mountain. He struck his pick into the pocket and the metal part of it turned to gold, and the sand he threw in turned to gold! Now he could forget his thirst—his hunger—and the blistering heat. Now he could die in peace! He was rich; he had the *power* to grow gold; that is, he thought he had, and so he died.

This old miner forgot that the rocks have long since ceased to grow. No, the mother lode for gold has never yet been found. But a greater mother lode is to be found in service to humankind!

Co-operation and service are two of the greatest principles in business today.

Well it has been said that all the great principles can be stated in a few words, they need no wordy embellishments, but—witness John Ruskin's essays—it does take much conversation sometimes to prepare the mind, to raise it to a level so that over a new horizon can be seen a sparkling star, a diamond of thought.

Let us look over the horizon of our own surroundings and find those favorable stars of thought and purpose which shall guide us to greater service to mankind, and result in greater happiness and prosperity.

Go back a moment to the dawn of civilization. Originally man lived by instinct alone; his needs were few; he lived in a cave and slew for food and protection. If one savage had corn, and his neighbor had sheep, any business transaction in either commodity had to be negotiated with a stone hatchet.

Slowley the human mental process developed the art of barter or exchange, and later of manufacture; first, exchange for immediate, pressing necessities, and later for *gain*—to have something in store so that necessities could be purchased during periods of idleness or travel.

At first, all industry was individual, then by families; next, slaves were compelled to co-operate in production, until today evolution has produced factories, corporations and trusts, and such things as scientific management.

Civilization had its birth as an association of races of men. Unconsciously, they grouped themselves together in co-operation to wield sufficient physical power to protect members against the invasions of other men, of wild beasts, and of the elements; also to accumulate information for use in the invention and production of human needs and comforts; and finally, to advance and to uphold above every other passion the love of justice and right, high moral standards—so that all men might have and enjoy in peace, the fruits of physical and mental effort.

The purpose of civilized association, in fact, of every organization of man, is the generation and accumulation of power—power to plan and to enforce a premeditated conclusion—whether the object be good or evil.

Let us look more carefully and divide this power into its kinds and varieties.

First: There is physical power—the power of a man's right arm and fist—the power of the waterfall that turns the mill—the power of a world in motion.

Second: There is a mental power. You have seen the strong forced by mental dominance to do the will of the physically weak, whether the purpose was good or ill. If the world turning on its orbit, exerts great physical force, think of the superior power of that Great Mentality that planned the world, and then forced it to swing by the word of His power.

Third: There is a moral power. It is the greatest of all. It is the thing that keeps the universe in order. It is the spring from which continuity and endurance flow—it is everlasting. Its standard is perfection. Its banner, displayed to humankind reads that "Right is mightier than might." It is faith in justice, faith in the triumph of good thoughts, good words and good deeds. If a man has faith that his principles or practices, are right, you know that no amount of pressure, mental or physical, can make him change. He may submit for a moment to physical torture or to mental fatigue, but change, never!

I have heard of men, and even seen some, who backed by moral power, have faced overwhelming odds, both physical and mental, and I have seen them out-talked and out-thought, yet they stood pedestalled high—their silence proof of their courage.

This moral power is the force used by Almighty God.

In the wielding of His great power is the principle of co-operation. It is the law of compensation—the law of action and re-action.

Co-operation in nature is the yielding to the law of attraction and repulsion. In these gravitations are involved the basic principle of service.

Men of science have found that the moon influences our earth—it serves us, and we serve it in return. The remotest star sends to us its living vibrations and we serve it by setting up a counter attraction. So, the universe is balanced in harmony by the co-operative service of its members. The smallest atom has its field of service in preserving the continuity of the universe. Even as you and I.

Gentlemen, the word "co-operation" includes the ideas of "power" applied to "service." Co-operation is the greatest word in business today. Every business and every business man must co-operate; retailer with wholesaler; competitors must co-operate between themselves, and the dentist, the physician, and the banker with them all; labor with capital; nation with nation. If we expect to uphold, to progress, and to develop our present civilization—political, as well as industrial—*we must know ourselves*, and, *we must know one another* in the deepest and truest meaning.

I was very much surprised, several years ago, to learn that a great eastern railroad purchases the oil for its signal tower lamps, not—as is usually the case—by the gallon, but at so much per hour for light; that is, it pays according to the service rendered by the oil.

Now, every individual in the world is measured or judged by the same standard. A banker must possess ability acquired through years of experience and study. The mastery of the technique of his profession creates the capacity for service. But the value of the service depends —not alone upon capacity—but upon the exercise of ability—ability that should be applied to the problems of the day with promptness, exactness and courtesy.

The value of service depends, I say, like the light in the railroad's signal tower, upon its dependable continuity!

Would you like a word regarding present business conditions?

One does not have to be a financier to be aware of the situation in business today. Since the beginning of the world war, the abnormal demand for all commodities increased business in this country many fold. Prices rapidly rose, until in May of this year when the peak of prices was reached, we were paying on an average for all commodities 178 per cent. above normal.

Many individuals and new concerns started in business making profits without much effort and certainly, in many instances, without much, if any, business ability.

Through this period of prosperity the old, experienced and wellmanaged concerns, realized that the day would come when a readjustment in prices must take place, and made provisions accordingly. Today, we are confronted with this situation—a falling market—and someone must suffer the loss.

About a year ago, financiers throughout the country foresaw that a change was about to take place, and to those who would accept it, the banker gave advice to use caution—buy lightly—liquidate.

Those who did not heed the warning are the ones to suffer. Statistics show that since the turning point in May there were 2,589 failures against 1,838 for the same period last year. The total liabilities of the failures for these periods were $111,416,000 this year against $29,838,000 last year.

This does not mean that all businesses will find themselves with insurmountable problems. All well-managed concerns will continue and be in a healthy financial condition after the period of readjustment.

Accompanying conditions such as this, there is always a serious financial situation to be met, necessitating careful study on the part of the bankers. With the higher price level, a greater amount of capital is required to conduct business—consequently, larger loans from the banks.

Banks throughout the country have found it necessary to call upon the resources of the Federal Reserve System for large sums in the form of rediscounts. At the same time, restrictions were put in force to conserve the country's financial strength—suggestions were made by the Federal Reserve Board to confine advances mostly to enterprises having to do with the manufacture and handling of essential commodities—food, clothing, shelter.

The financial situation at the beginning of this year was looked upon as very serious; in fact, a crisis was fully expected this fall. I figured that about October 15th we would see the peak of the situation. But, I am happy to say the first of October saw the clouds of disaster clearing away very rapidly. The falling market prompted liquidation, merchants throughout the country thought best to sell while the prices were high and offered inducements to the public—taking their loss—and with the money realized from these sales, reduced materially their obligations to the banks.

I have reviewed the business condition for you for one reason in particular—that is, to show you the necessity of co-operation between the business man and the banker. If the business men can benefit by having the banker confer with him on his problems, why should not the individual also who is not directly interested in business benefit as well?

But, the individual requires a different kind of cooperation than the business man. What is necessary in his case, is advice, information, service.

The knowledge that a banker obtains through constant contact with business, financial and economic conditions, is in itself of great value, to the vast majority of those not directly in touch with these affairs.

Men in your profession, as a class, concentrate so thoroughly upon your professional duties that if any one should become a good business man, it is usually natural talent rather than education.

Recently several instances came to my attention where investments by professional men were made unwisely. Doctors do not always invest wisely. This is because they can analyze proportions that are fairly good and are sharp enough to be cautious about matters they can understand, but investments that they can not understand they usually buy without analysis because they believe in the salesman or someone else's ability to judge. Almost every share of mining and oil stock that doctors buy is purchased without consideration of the antecedents, reputation, character, or efficiency of the men and organizations offering securities.

Many changes are taking place in business today, and quite naturally it is having its effect on investments.

The business of the banker is to know about investments, just as it is the business of the dentist and physician to know about disease and the means of preventing and remedying human ills.

The three common means of investment are:

1. Directly in some private enterprise.
2. Purchase of stocks and bonds.
3. Mortgages.

In deciding upon an investment, one must aberrate between the different characteristics—merit on the one hand and deficiency on the other—making final judgment between the qualities of the securities and the requirements of the investor.

Let me quote, from an authority on this subject, the five chief points to be considered in the selection of an investment:

1. Security of principal and interest, or the assurance of receiving the principal and interest when due.

2. Rate of income, or the net return which is realized on the actual amount of money invested.

3. Convertibility into cash with which is included availability as collateral.

4. Minimum fluctuation, or stability of market price.

5. Prospect of appreciation in value.

With the numerous sources of information which a bank has, it is a comparatively easy matter for them to make the necessary investigation in connection with contemplated investments and to furnish reports upon which to base a decision.

Not only should investigation be made before purchasing stocks or bonds, and in making other investments, but they should be checked up periodically.

There are many other ways also in which your banker might render a valuable esrvice, *but if you do not cultivate him you will never benefit by his co-operation.*—By Mr. C. W. Banta, San Francisco, Cal., in *Pacific Dental Gazette* of January, 1921.

